# Supplementary material for: A Pilot Study on the Effects of Transcranial Direct Current Stimulation on Brain Rhythms and Entropy during Self-Paced Finger Movement using the Epoc Helmet
Source: Front Hum Neurosci. 2017 Apr 25;11:201. doi: 10.3389/fnhum.2017.00201 (PMC5408787; doi:10.3389/fnhum.2017.00201)
Supplement: Supplementary file 1 [file Data_Sheet_1.zip › Complementary_results/Kullback-Leibler-Divergence_Basal-Referenced_Complete_EEG/Kullback-Leibler_Basal-Referenced_Complete-EEG_F7.pdf]

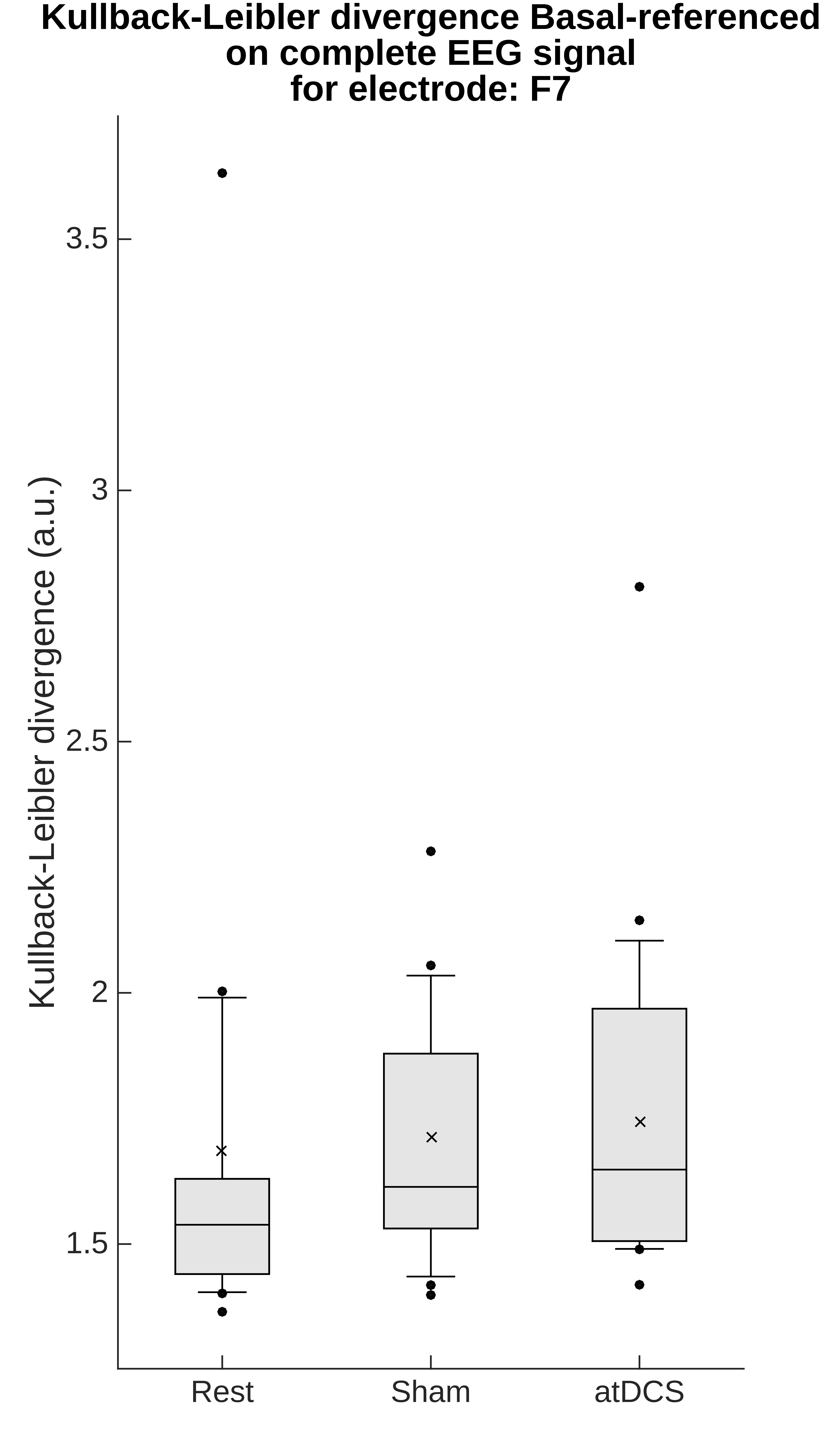
Kullback-Leibler divergence Basal-referenced
on complete EEG signal
for electrode: F7
Kullback-Leibler divergence (a.u.)
3.5
3
2.5
2
1.5
Rest
Sham
atDCS
